# Supplementary material for: A conserved protein inhibitor brings under check the activity of RNase E in cyanobacteria
Source: Nucleic Acids Res. 2023 Nov 24;52(1):404–19. doi: 10.1093/nar/gkad1094 (PMC10783494; doi:10.1093/nar/gkad1094)
Supplement: gkad1094_Supplemental_Files [file gkad1094_supplemental_files.zip › Supporting Information.pdf]

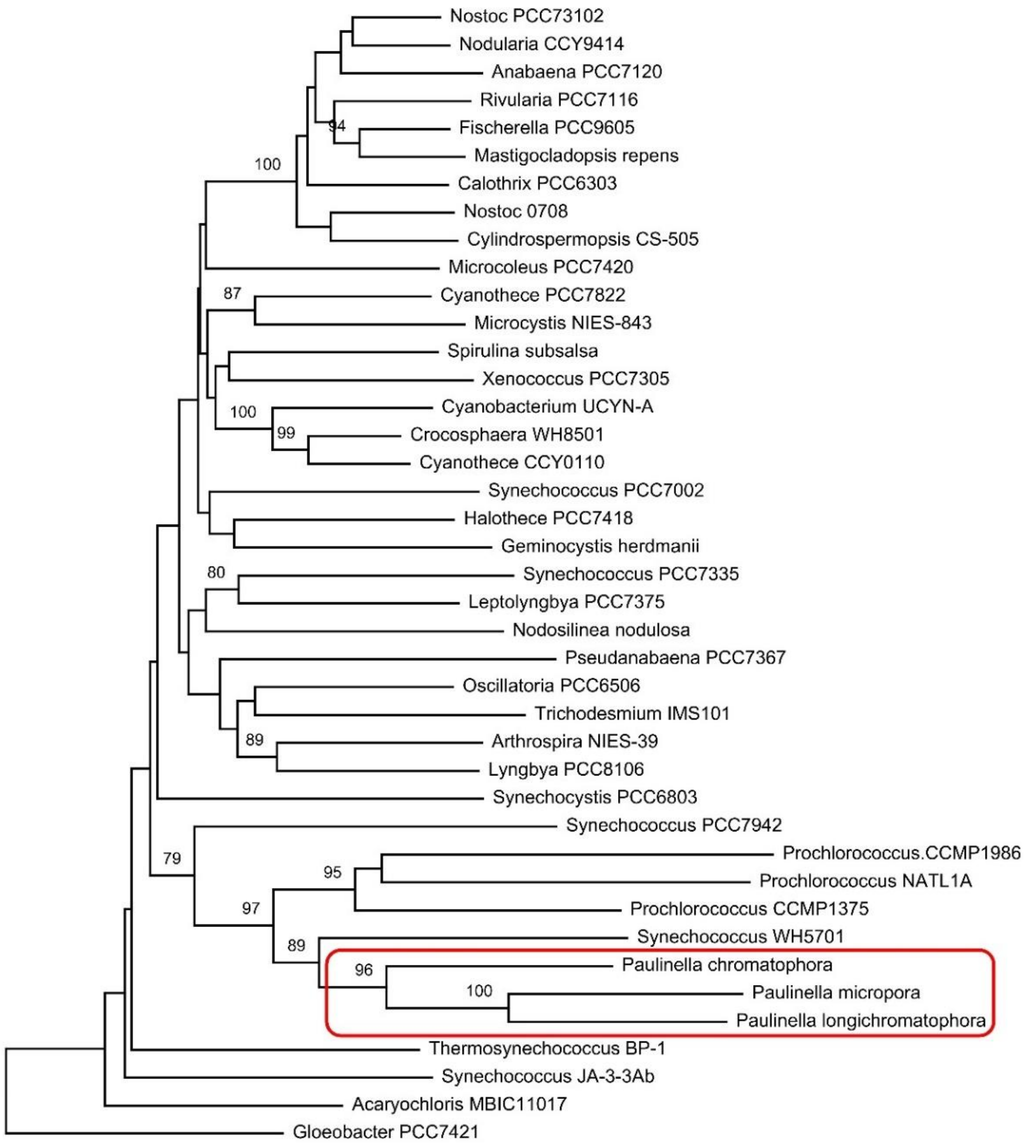

Figure S1. Evolutionary relationships of RebA homologs. The sequences of RebA homologs were retrieved from GenBank followed by a BLASTP search using RebA of *Anabaena* PCC 7120 as the query. The sequences from 38 cyanobacterial strains that could represent the diversity of cyanobacterial species (1) and 3 non-cyanobacterial strains of *Paulinella* were selected for phylogenetic analysis. These sequences were aligned and analyzed using MEGA X (2). The evolutionary history was inferred using the Neighbor-Joining method (3). Numbers above the branches are the percentage of replicate trees in which the associated taxa clustered together in the bootstrap test (500 replicates) (4). Only the percentage values higher than 75% are shown.

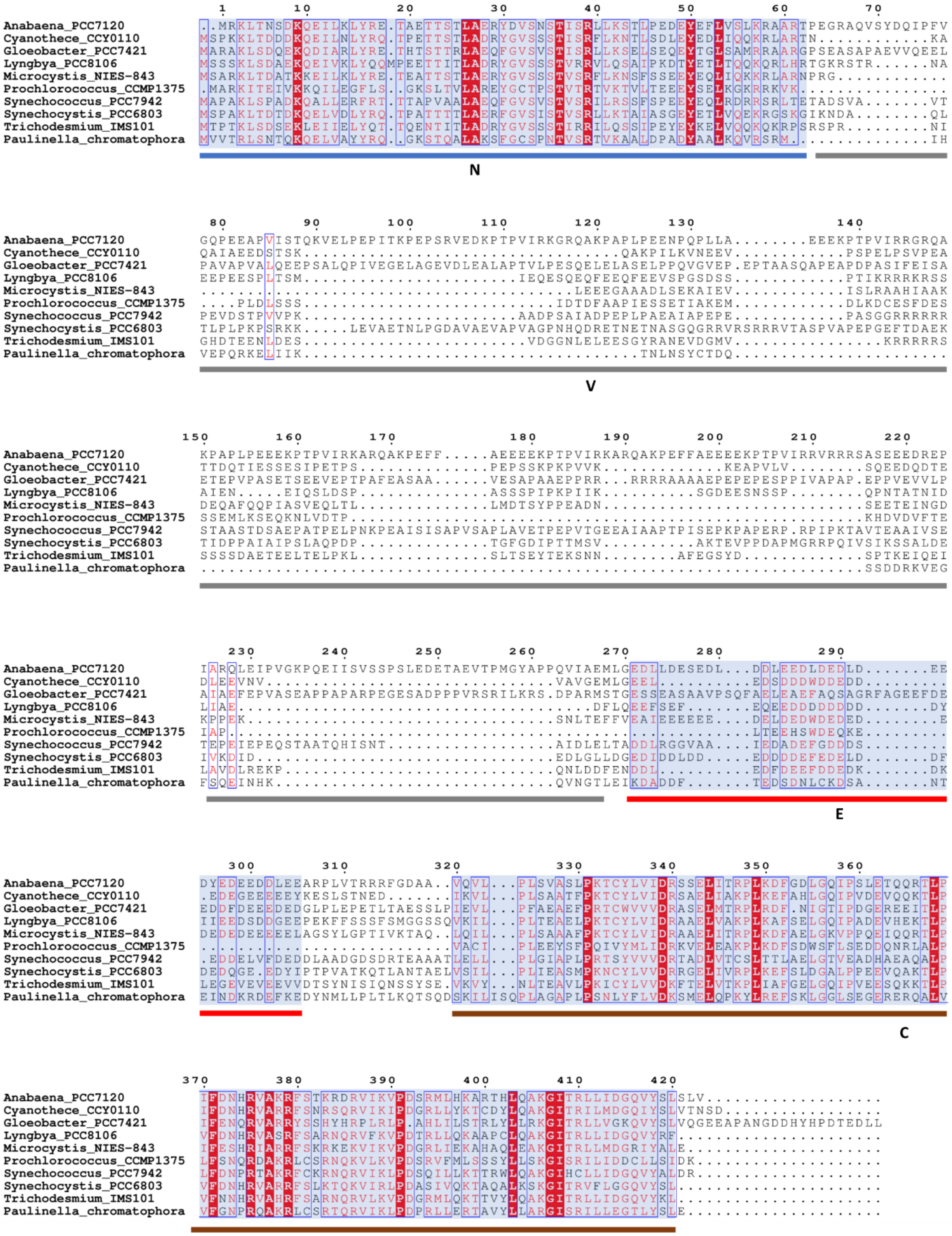

Figure S2. Sequence alignment of RebA homologs from several phylogenetically distant cyanobacterial species and *Paulinella*. The alignment was created in MAGA X (2). Alignment formatting was performed in ESPript 3.0 (5) with the default parameters. Residues with red background are completely conserved while those in red are semi-conserved. Sequence numbering is relative to *Anabaena* RebA. Four domains are defined based on the alignment of RebA homologs: N and C, the conserved regions at the N-terminal and the C-terminal, respectively; V, the highly variable region; E, the region rich in glutamic acid and aspartic acid.

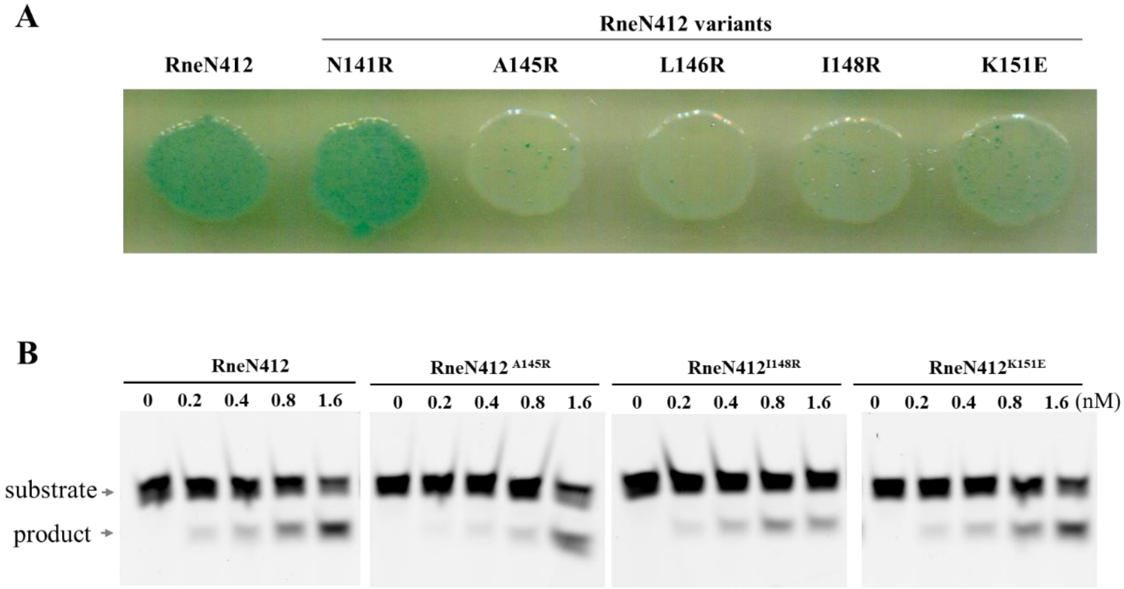

Figure S3. Key residues within the 5' sensor domain of RNase E required for RebA-RNase E interaction. (A) Bacterial two-hybrid assay testing the interactions between RebA_C and several variants of the catalytic domain of RNase E (RneN412) that contain point mutations within the 5' sensor domain. (B) The cleavage of 5' p-LU13-FAM by the RneN412 variants $RneN412^{A145R}$, $RneN412^{I148R}$, and $RneN412^{K151E}$. RneN412 was used as a positive control, respectively. Each reaction contained 50 nM 5' p-LU13-FAM and indicated amount of enzyme. After 20 minutes of incubation at 30°C, the reactions were stopped and analyzed by 15% urea-PAGE.

A

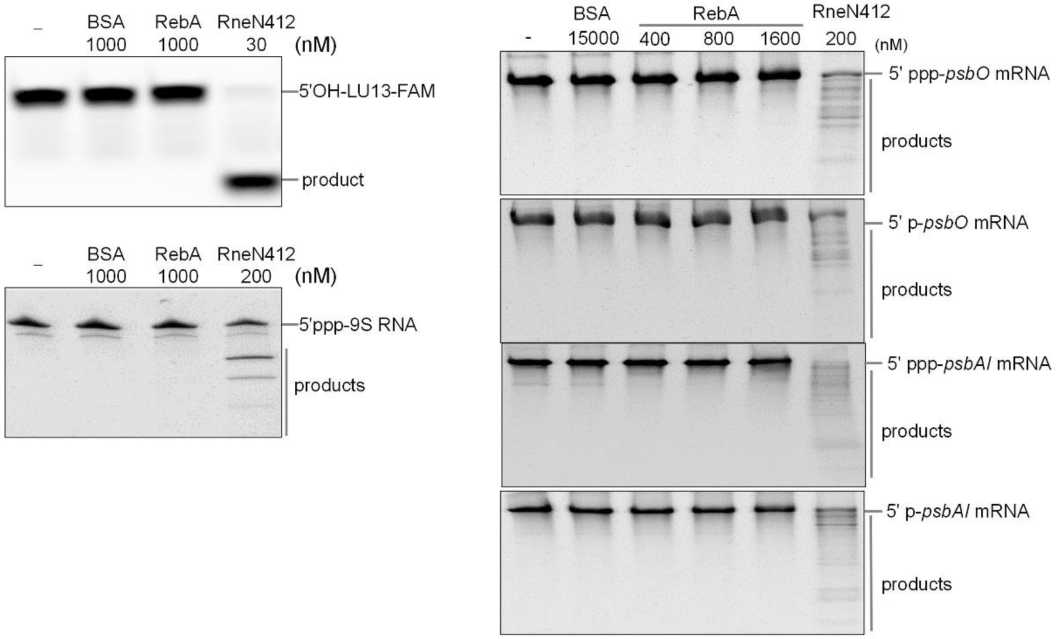

B

***psbAI* mRNA (1209 nt)**

GACAGGCGCAAGUCUAAAAAAAAGCGUCUGAAUUAAUCUGCACAAAUCCAAAGCAAUCAUAAAAAA**AUG-(1077nt)-UAA**UCUUCAGUUUUAGUUAACUAAAAAAGCGCCCUUCCAAUUUGCAUGGAAGAGCGCUUUUUUA

***psbO* mRNA (1314 nt)**

GAUUUCCCUAAUAAAGCAAGCGGCUGUAUUUAGUGGUAACACAUACAUAAUACAGAACGACUUGGGCAACC
CUGCCAACACCACAGGUAUUGGCACAGCGCGAAGCCAAAUCCAACUUUCGCGUUAUUUGAGGUAAGUAGU
GAUGCCAAUUUGCCGCAAAAUGUACUUAUCACUAUGCCCAAACCCUUUAUAUAUCGAUUUUUGACAUUGAG
GUUGAUC**AUG-(816nt)-UAA**ACCCAAACGUGCCUAAAUUUUCUUGAUUGCCAAAGCUGUCUGGCACUGGAAC
CAUUCUUUGCCUAUUAUCAAAUUUCUGAUUGAACGUUAACCCAUACCCCCUAAUUUUCCUGAGGUGCAUGG
UUUGACUACUCGCUUAACUAACGCAAUUUUCGGGUUGAUAAAAUCCCGUGAGGACAGGGAGUUGGAAAA
AUGCAGUUCAAUCAGUGGUAAAAAGCUGUUAAUACUCUCAAAAAAAAAUCACAGAAAGGGGCUUCUAGCCCU
UUUUUUAUU

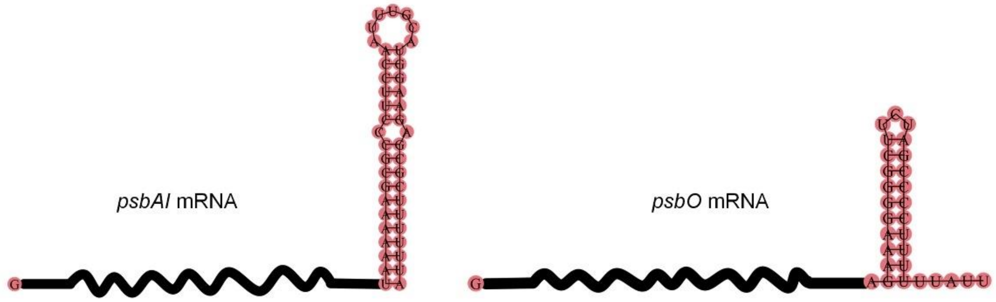

Figure S4. Testing the RNA cleavage/degradation activities of RebA on various RNA substrates. (A) RebA showed no cleavage/degradation activities on 5' OH-LU13-FAM, 5' ppp-9S RNA, *psbO* mRNA and *psbAI* mRNA (either with 5' p or 5' ppp). Each substrate was incubated with RebA in the reaction buffer, with BSA and RneN412 being used as a negative control and a positive control, respectively. In the reactions, 5' OH-LU13-FAM were used at 50 nM, 5' ppp-9S RNA, *psbO* mRNA and *psbAI* mRNAs were used at 500 nM. BSA, RebA and RneN412 were used at

concentrations as indicated for each substrate. After 30 minutes of incubation, the reactions were stopped and analyzed by urea-PAGE. Substrates alone were loaded in the leftmost lanes (－) of the gels. (B) Sequence and cartoon representation of *psbA1* and *psbO* mRNAs used in this study. The initiation bases (in red) of these mRNAs were determined experimentally (6). The termination regions (in green) were predicted as they have features typical of a Rho-independent terminator (i.e., a stable stem-loop followed by a series of uridine residues). The termination residues that can form a stem by base-paring are underlined. The residues within the ORF regions are not shown for brevity.

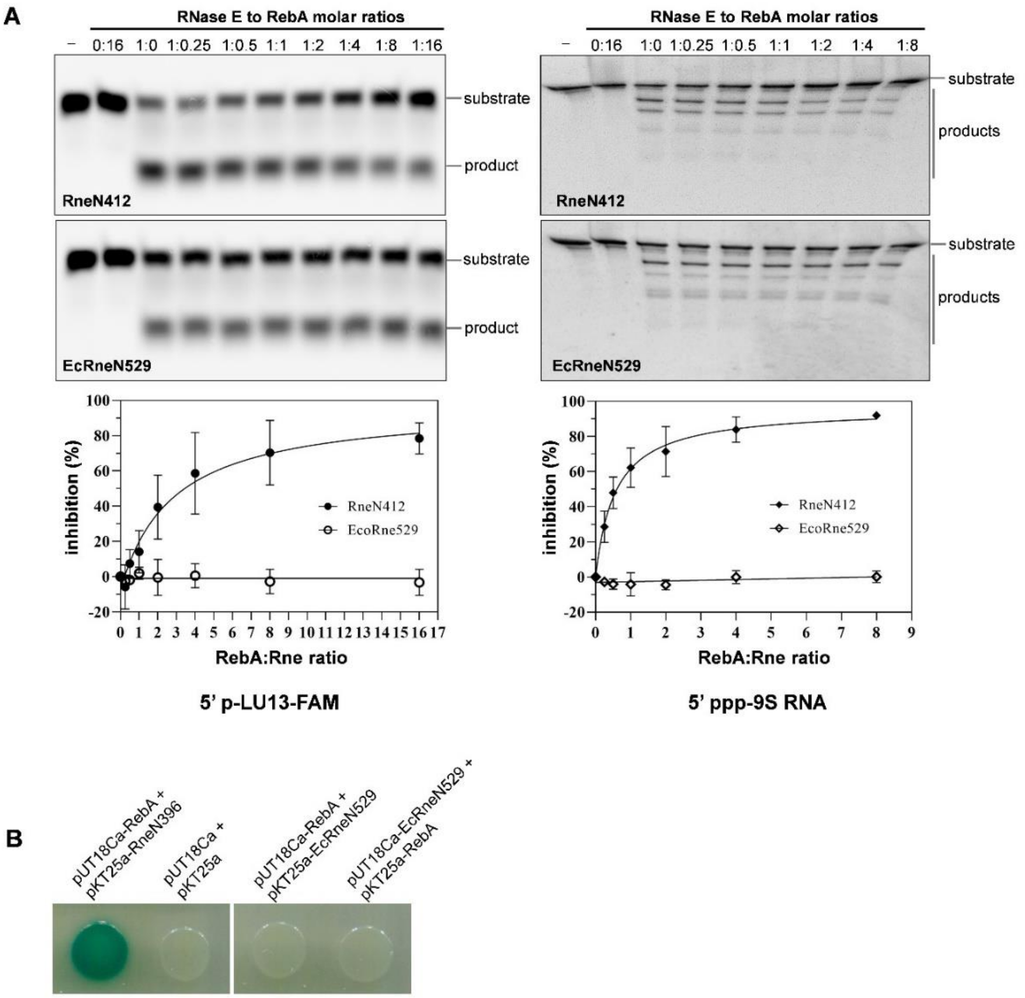

Figure S5. Enzymatic and bacterial two hybrid assays showing that RebA neither inhibits the activities of nor interacts with *E. coli* RNase E. (A) Comparing the inhibitory effect of RebA on the cleavage activity of the catalytic regions of *Anabaena* RNase E (RneN412) and *E. coli* RNase E (EcRneN529) using the substrate 5' p-LU13-FAM and 5' ppp-9S RNA. The assay conditions were identical to those used in Figure 2D. (B) Testing the interaction between RebA and EcRneN529 using bacterial two hybrid assay. The reporter strain containing the plasmids of pUT18Ca-RebA and pKT25a-RneN396 was used as a positive control and that containing the empty vectors of pUT18Ca and pKT25a was used as a negative control.

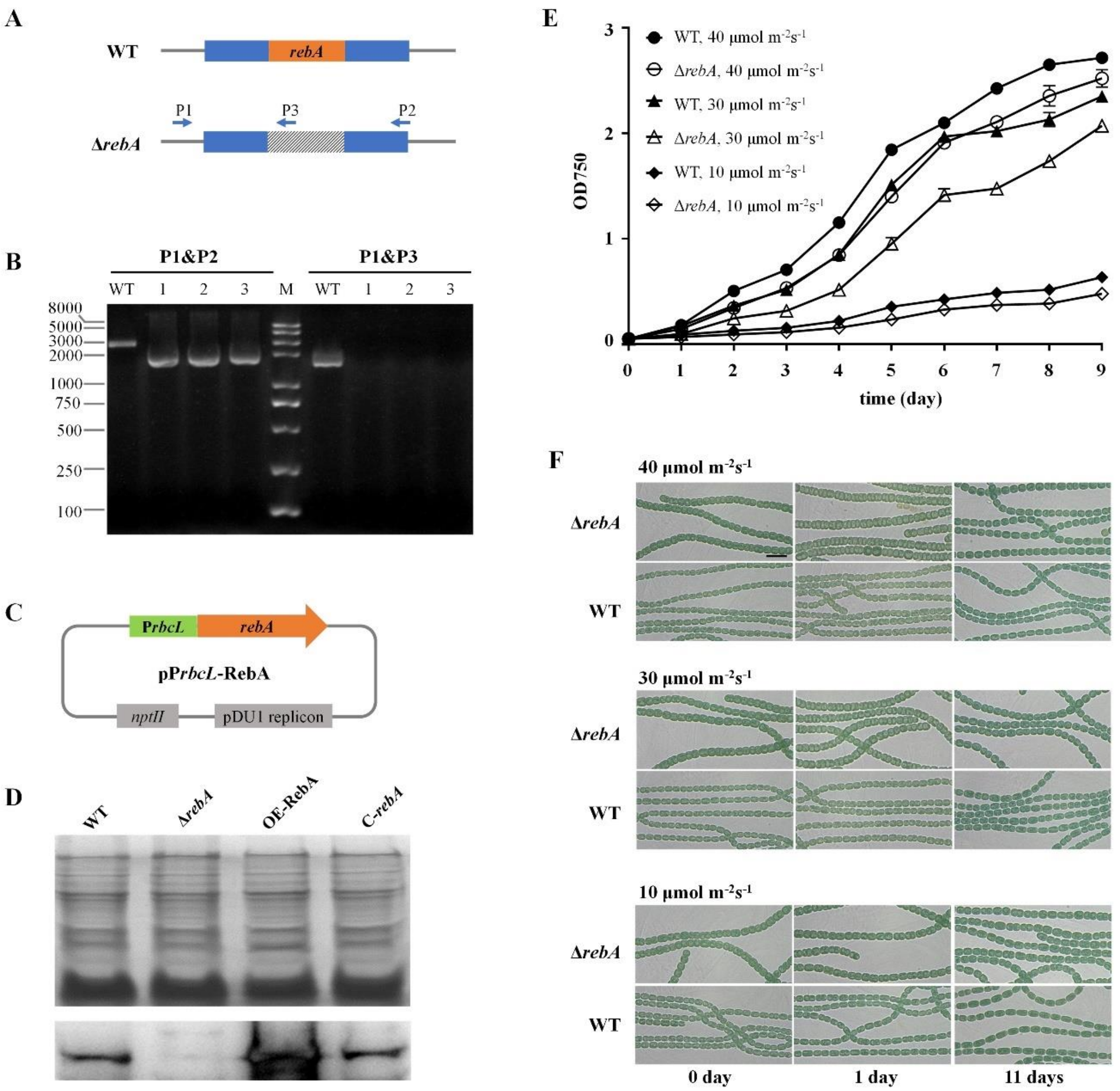

Figure S6. Construction of *Anabaena rebA* mutation strain and overexpression strain, and checking the growth and cell morphology of the *rebA* mutant strain under different light intensities in BG11 medium. (A) Schematic representation of the genotype of *Anabaena* wild type strain (WT) and the mutant strain of *rebA* (Δ*rebA*). (B) Verification on the genotype of Δ*rebA* by PCR using the primers shown in panel A (arrows). P1, P2, and P3 are short names for the primers of Pall1340F681, Pall1338R2225, and Pall1338R797, respectively (sequences listed in Supplementary Table S3). Three colonies from the conjugation plates were examined. With P1 and P2, the expected sizes of the PCR products for WT and Δ*rebA* would be 3313 bp and 2086 bp, respectively. With P1 and P3, there would be a 1871 bp product for WT and no product for Δ*rebA*. (C) Schematic representation of the replicative plasmid pP*rbcL*-RebA, which was transferred into WT to obtain the RebA overexpression strain OE-RebA and into Δ*rebA* to obtain the complementation strain (C-*rebA*). Only the main features of the plasmid are shown. P*rbcL*, the promoter of the *Anabaena rbcL* gene that encodes the large-subunit of ribulose bisphosphate carboxylase; the promoter is a relative strong and often used for gene overexpression in *Anabaena*; *rebA*, the ORF of *rebA* gene; pDU1 replicon, the region required for plasmid replication in *Anabaena*; *nptII*, the neomycin resistance gene. (D) Comparing the expression of RebA in the strains of WT, Δ*rebA*, the overexpression strain OE-RebA and the complementary strain C-*rebA* by Western blot. The protein samples of the strains were loaded at 50 μg per lane onto duplicates of SDS-PAGE

gels, with one gel stained with Coomassie brilliant blue staining (upper) and the other immunodetected with the polyclonal antibodies against RebA (lower). (E) Growth curves of Δ*rebA* and WT under high light (40 μmol $m^{-2}s^{-1}$), medium light (30 μmol $m^{-2}s^{-1}$) and low light (10 μmol $m^{-2}s^{-1}$) conditions. (F) Microscopic images of the Δ*rebA* and WT cells under the indicated light conditions. The shorter-cell phenotype of Δ*rebA* appeared when cell density was relatively low and light intensity was relatively high. The samples for microscopy were taken at the indicated time points during the process of growth curve measurement (panel E). Scale bar: 10 μm.

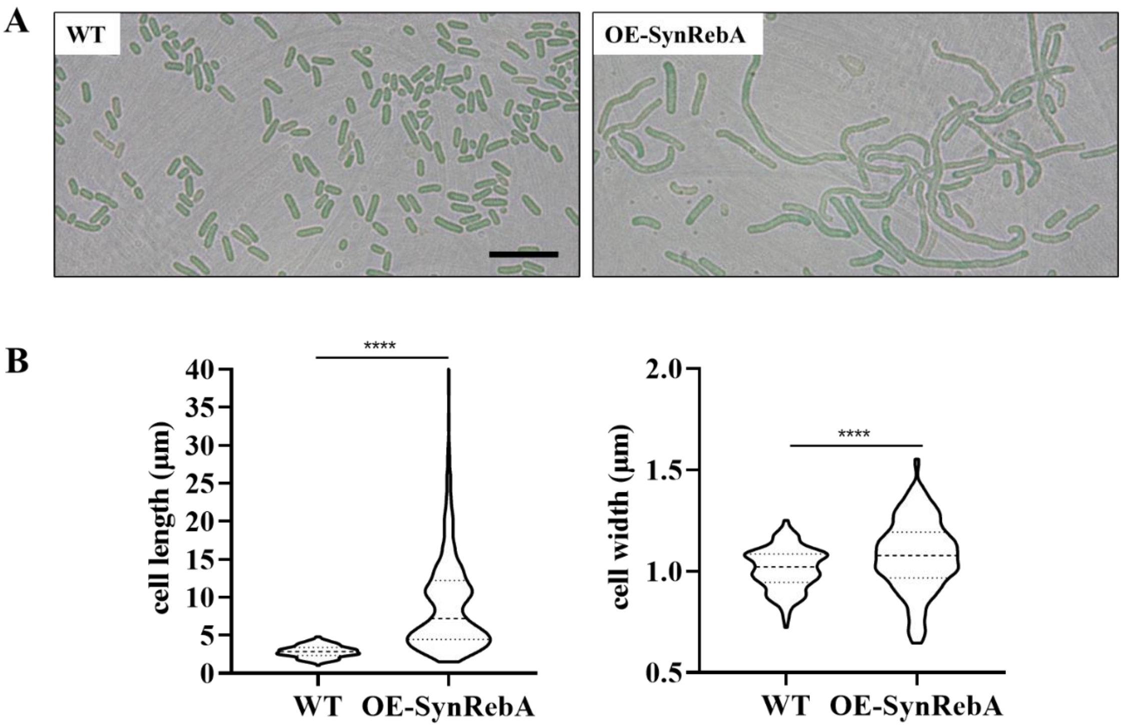

Figure S7. Effect of RebA over expression in *Synechococcus elongatus* PCC 7942. (A) The RebA-overexpression strain (OE-SynRebA) contains a replicative plasmid carrying an extra copy of the native *rebA* homolog gene. The strains were cultured in liquid BG11 medium under the standard light condition (30 μmol m-2s-1) and imaged when the culture density reached OD700 = 0.5. Scale bar: 10 μm. (B) Distribution of the cell lengths and the cell widths of the cells from WT and shown in panel A. A total of 200 cells from each strain were measured and quantified by ImageJ. The violin plotting and statistical analysis of data were performed with the GraphPad Prim software. P values were calculated using the nonparametric Mann-Whitney test in comparison to WT. Significant P-values are marked by ****P < 0.0001.

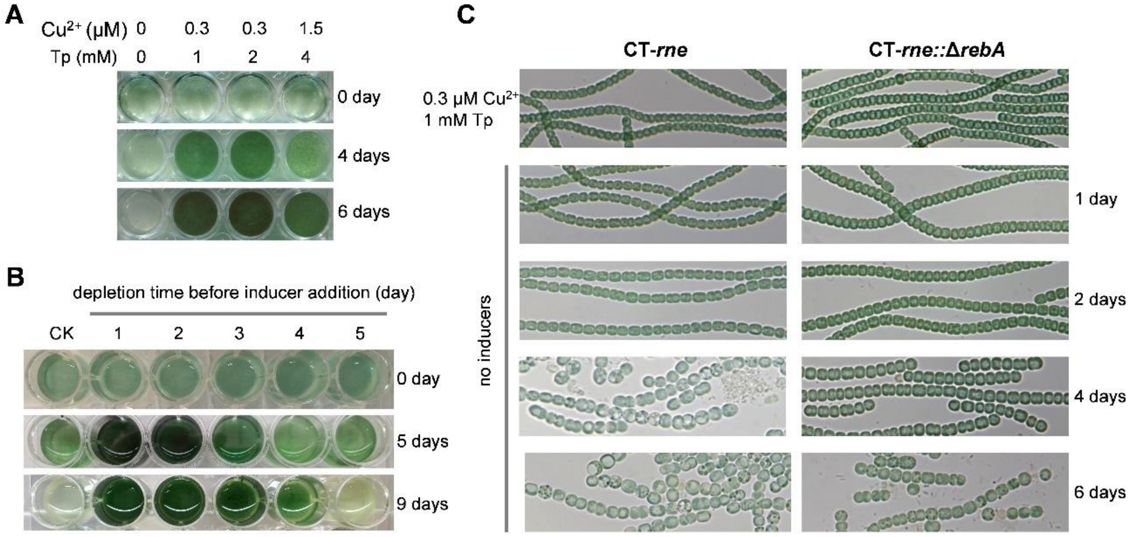

Figure S8. Cell viability of the *rne* conditional mutant CT-*rne* under different induction conditions and the effects of *rebA* inactivation in CT-*rne*. (A) Growth of CT-*rne* cells in the BG11 media with different concentrations inducers. (B) Viability of CT-*rne* cells after RNase E depletion in the medium without inducers. CT-*rne* cells precultured in the BG11 medium containing 0.3 μM Cu^{2+} and 1 mM theophylline were inoculated into the wells of a 24-well plate containing the inducer-free medium with the initial density of OD700 ≈ 0.3. After indicated time periods of growth without inducers, 0.3 μM Cu^{2+} and 1 mM theophylline were added into the wells. No inducers were added into the CK well. The cultures were grown in 24-well plates under normal condition (i.e., shaking speed of 180 rpm, light intensity of 30 μmol $m^{-2}s^{-1}$, and 30 °C). (C) The effects of *rebA* inactivation in CT-*rne*. The microscopic images of CT-*rne* and its derivate strain CT-*rne*:: Δ*rebA*, in which *rebA* was additionally inactivated with the plasmid pCpf1b-ΔrebA, were compared. The strains were first cultured in BG11 medium containing 0.3 μM Cu^{2+} and 1 mM theophylline (Tp), then transferred into the BG11 medium without the inducers for RNase E depletion. CT-*rne* cells became significantly longer after two days of RNase E depletion, and severe cell lysis and filament fragmentation occurred after four days of depletion. In contrast, when *rebA* was deleted in CT-*rne*, cell morphology change, filament fragmentation and cell lysis were significant delayed after RNase E depletion.

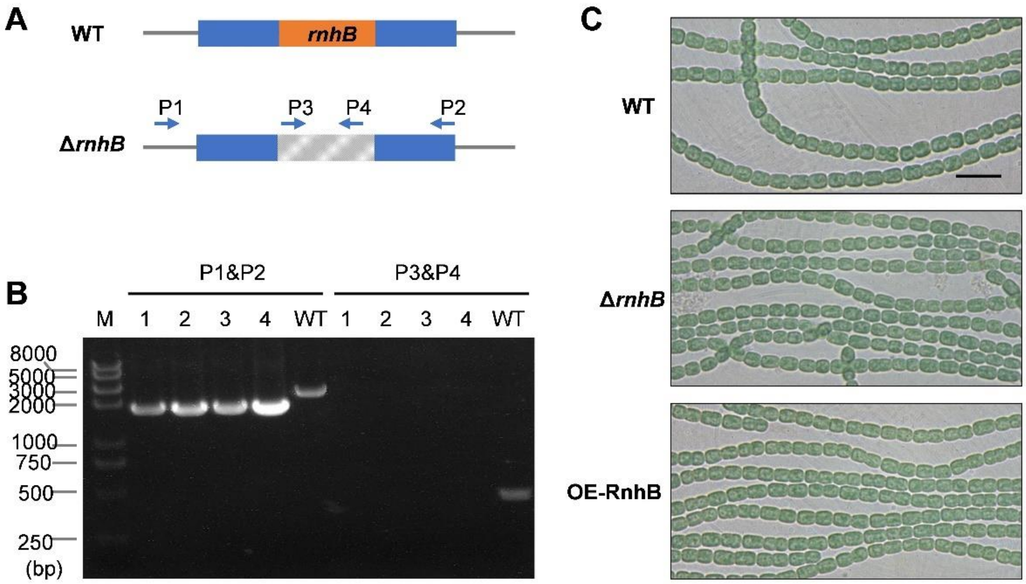

Figure S9. Inactivation of *rnhB*, the gene downstream of *rne* did not affect cell morphology. (A) Schematic representation of the genotype of *Anabaena* wild type strain (WT) and the mutant strain of *rnhB* (Δ*rnhB*). (B) Verification on the genotype of Δ*rnhB* by PCR using the primers shown in panel A (arrows). P1, P2, P3, and P4 are short names for the primers of Palr4332F928m, Palr4332R1538, Palr4332F179 and Palr4332R586, respectively (sequences listed in Supplementary Table S3). Four colonies from the conjugation plates were examined. With P1 and P2, the expected sizes of the PCR products for WT and Δ*rnhB* would be 2466 bp and 1788 bp, respectively. With P3 and P4, there would be a 437 bp product for WT and no product for Δ*rnhB*. (C) Comparison of the microscopic images of the filaments of WT, Δ*rnhB* and the RnhB overexpression strain OE-RnhB. The samples for microscopy were taken from fresh cultures (OD700 ≈ 0.3) grown in BG11.

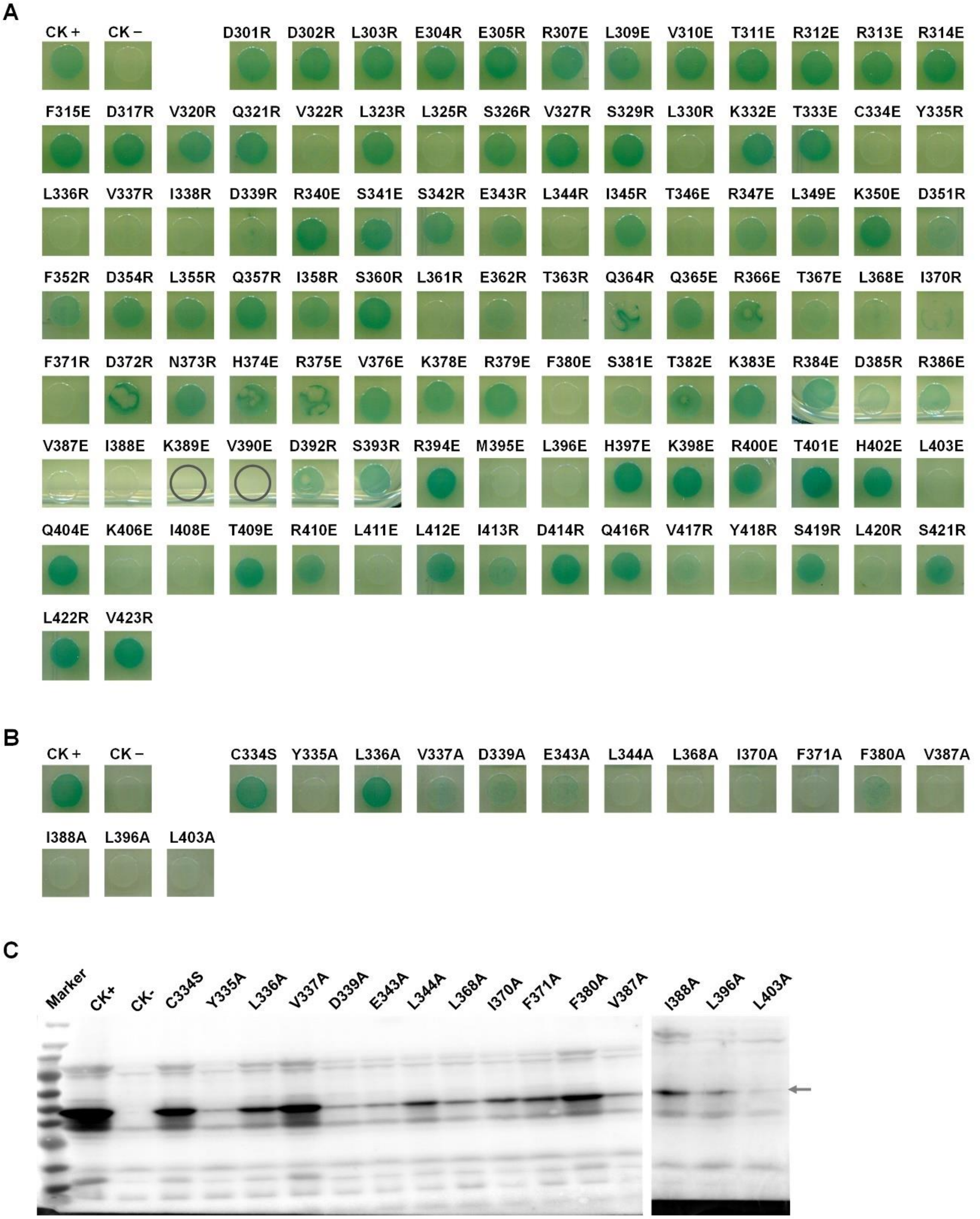

Figure S10. Screening for the key residues required for RebA-RNase E interaction by site-directed mutagenesis and bacterial two hybrid assays. Two rounds of screening were performed. (A) The first round of mutation screening. The residues of alanine, glycine and proline within RebA_C were not changed as they are unlikely involved interaction or their mutation may disrupt the protein structure. Creation of the mutations of K389E and V390E was not successful (indicated by circles). CK+ and CK-, the *E. coli* strains co-transformed with pUT18Ca-RneN396/pKT25a-RebA_C and pUT18C/pKT25, respectively. The other strains contained pUT18Ca-RneN396 and pKT25a-RebA_C derivates expressing the indicated RebA_C variants. (B)

The second round of mutation screening. The sites whose mutation had showed significantly decreased interaction signal in the first round of screening were further changed into the small neutral residues of serine (for cysteine) or alanine (for other residues). (C) Confirming the expression of RebA_C variants in the two-hybrids strains used in panel B by Western blot. The antibodies against RebA were used. Arrow indicates the bands of RebA_C and its variants.

Table S1. Cyanobacterial and *E. coli* strains used in this study. Abbreviations: Nm^r, neomycin resistance; Amp^r, ampicillin resistance; Cm^r, chloramphenicol resistance.

Strains	Description	Source
Anabaena sp. PCC 7120 (WT)	the wild type strain of *Anabaena* sp. PCC 7120	R. Haselkorn
Synechococcus elongatus PCC 7942	the wild type strain of *Synechococcus elongatus* PCC 7942	Pasteur Culture collection of Cyanobacteria (PCC)
OE-RebA	WT bearing the replicative plasmid pP_{rbcL}-RebA; Nm^r	this study
OE-SynRebA	*Synechococcus elongatus* PCC 7942 bearing the replicative plasmid pCT-SynRebA; Nm^r	this study
Δ*rebA*	A markerless deletion mutant strain of *rebA*	this study
C-RebA	Δ*rebA* bearing pP_{rbcL}-RebA; Nm^r	this study
CT-*rne*	*rne* conditional mutant strain, in which the native promoter of *rne* is replaced with the inducible CT promoter	this study
CT-*rne*::Δ*rebA*	CT-*rne* with additional deletion of *rebA* gene from the chromosome	this study
OE-$RebA^{V337A}$	WT bearing the plasmid pP_{rbcL}-$RebA^{V337A}$; Nm^r	this study
OE-$RebA^{I368A}$	WT bearing pP_{rbcL}-$RebA^{I368A}$; Nm^r	this study
OE-$RebA^{I370A}$	WT bearing pP_{rbcL}-$RebA^{I370A}$; Nm^r	this study
OE-$RebA^{V387A}$	WT bearing pP_{rbcL}-$RebA^{V387A}$; Nm^r	this study
OE-$RebA^{I388A}$	WT bearing pP_{rbcL}-$RebA^{I388A}$; Nm^r	this study
Δ*rnhB*	a markerless deletion mutant strain of *rnhB*	this study
OE-RnhB	WT bearing pCT-RnhB; Nm^r	this study
BL21(DE3)	*E. coli* host strain for recombinant protein production	WEIDI Bio (CAT#: EC1002)
BTH101	*E. coli* host strain (*cya*$^-$) for bacterial adenylate cyclase two-hybrid assay	7
DH10B (pRL443+pRL623)	*E. coli* strain used to transfer cargo plasmid into cyanobacteria by conjugation, containing the conjugal plasmid pRL443 and the helper plasmid pRL623; Amp^r, Cm^r.	8

Table S2. Plasmids used in this study. Abbreviations: Km^r, kanamycin resistance; Nm^r, neomycin resistance; Sm^r, streptomycin resistance; Sp^r, spectinomycin resistance; Car^r, carbenicillin resistance.

Plasmid	Description	Source
**for cyanobacteria strains construction**		
pCT	Nm^r; a pDU1-derived vector that contains the inducible CT promoter	9
pCpf1b-sp	Sp^r/Sm^r; CRISPR/Cpf1-based genome editing vector	10
pCpf1	Nm^r; CRISPR/Cpf1-based genome editing vector	10
pICT-rne	Sp^r/Sm^r; the genome editing plasmid for *rne* conditional mutation	this study
pCpf1b-ΔrebA	Sp^r/Sm^r; the genome editing plasmid for *rebA* markerless deletion	this study
pP_{rbcL}-RebA	Nm^r; pRL25N-derived plasmid for RebA expression under the relatively strong promoter P_{rbcL}.	this study
pP_{rbcL}-$RebA^{V337A}$	Nm^r; pP_{rbcL}-RebA derivate for expressing the RebA variant that has the V337A mutation	this study
pP_{rbcL}-$RebA^{I368A}$	Nm^r; pP_{rbcL}-RebA derivate for expressing the RebA variant that has the I368A mutation	this study
pP_{rbcL}-$RebA^{I370A}$	Nm^r; pP_{rbcL}-RebA derivate for expressing the RebA variant that has the I370A mutation	this study
pP_{rbcL}-$RebA^{V387A}$	Nm^r; pP_{rbcL}-RebA derivate for expressing the RebA variant that has the V387A mutation	this study
pP_{rbcL}-$RebA^{I388A}$	Nm^r; pP_{rbcL}-RebA derivate for expressing the RebA variant that has the I388A mutation	this study
pCpf1b-ΔrnhB	Sp^r/Sm^r; the genome editing plasmid for *rnhB* markerless deletion	this study
pCT-SynRebA	Nm^r; pCT carrying the *Synechococcus rebA* gene under the control of the CT promoter, used for RebA (Synpcc7942_0395) overexpression in *Synechococcus elongatus* PCC 7942	this study
pCT-RnhB	Nm^r; pCT carrying *rnhB* gene under the control of the CT promoter, used for RnhB overexpression	this study
**for recombinant protein expression**		
pET28a	Km^r; vector for expressing recombinant protein with an N-terminal His tag and/or a C-terminal His tag.	Invitrogen
pHStag	Km^r; pET28a-derived vector for expressing recombinant protein with an C-terminal Strep tag and/or a N-terminal His tag.	9
pNStrep	Km^r; pET28a-derived vector for expressing recombinant protein with an N-terminal Strep tag and/or a C-terminal His tag.	this study
pHTAlr4331N412	Km^r; for expressing the catalytic domain of *Anabaena* RNase E (1–412 aa) with an N-terminal His tag	11

pHTAlr4331N412^{A145R}	Km^r; same as pHTAlr4331N412, but for expressing the RNase E N-terminal region (1–412 aa) variant having the point mutation A145R	this study
pHTAlr4331N412^{I148R}	Km^r; same as pHTAlr4331N412, but for expressing the RNase E N-terminal region (1–412 aa) variant having the point mutation I148R	this study
pHTAlr4331N412^{K151E}	Km^r; same as pHTAlr4331N412, but for expressing the RNase E N-terminal region (1–412 aa) variant having the point mutation K151E	this study
pHTAlr4331N396	Km^r; for expressing the catalytic domain of *Anabaena* RNase E (1–396 aa) with an N-terminal His tag	this study
pHT-EcRneN529	Km^r; for expressing the catalytic domain of *E. coli* RNase E (1–529 aa) with an N-terminal His tag	this study
pNStrep-RebA	Km^r; for expressing RebA with an N-terminal Strep tag; the ORF of rebA was codon-optimized and cloned into the vector pNStrep.	this study
pNStrep-RebA^{V337A}	Km^r; same as pNStrep-RebA, but for expressing the RebA variant having the point mutation V337A	this study
pNStrep-RebA^{L368A}	Km^r; same as pNStrep-RebA, but for expressing the RebA variant having the point mutation I368A	this study
pNStrep-RebA^{I370A}	Km^r; same as pNStrep-RebA, but for expressing the RebA variant having the point mutation I370A	this study
pNStrep-RebA^{V387A}	Km^r; same as pNStrep-RebA, but for expressing the RebA variant having the point mutation V387A	this study
pNStrep-RebA^{I388A}	Km^r; same as pNStrep-RebA, but for expressing the RebA variant having the point mutation I388A	this study
**for bacterial two-hybrid assay**		
pKT25a	Km^r; vector for two-hybrid assay, encoding the adenylate cyclase fragment T25. Derived from pKT25 (7) by changing the cloning site for easier cloning.	this study
pUT18Ca	Car^r; vector for two-hybrid assay, encoding the adenylate cyclase fragment T18. Derived from pUT18C (7) by changing the cloning site of pUT18 to that of pKT25a, so that a same fragment can be cloned into both pKT25a and pUT18Ca.	this study
pKT25a-zip	Km^r; positive control plasmid used in bacterial two-hybrid assay	7
pUT18C-zip	Car^r; positive control plasmid used in bacterial two-hybrid assay	7
pKT25a-RebA	Km^r; plasmid derived from pKT25, encoding RebA fused to the C-terminal of T25.	this study
pUT18Ca-RebA	Car^r; pUT18Ca derivate encoding RebA fused to the C-terminal of T18.	this study
pKT25a-RebA_C	Km^r; pKT25 derivate encoding RebA_C (301-424 aa) fused to the C-terminal of T25.	this study
pUT18Ca-RebA_C	Car^r; pUT18Ca derivate encoding RebA_C (301-424 aa) fused to the C-terminal of T18.	this study
pKT25a-Alr4331	Km^r; pKT25 derivate encoding RNase E fused to the C-terminal of T25.	9

pUT18Ca-Alr4331	Car^r; pUT18Ca derivate encoding RNase E fused to the C-terminal of T18.	9
pKT25a-Alr4331N	Km^r; pKT25 derivate encoding RNase E N-terminal region (1-396 aa, the catalytic region) fused to the C-terminal of T25.	9
pUT18Ca-Alr4331N	Car^r; pUT18Ca derivate encoding RNase E N-terminal region (1-396 aa, the catalytic region) fused to the C-terminal of T18.	9
pUT18Ca-Alr4331N^{N141R}	Car^r; same as pUT18Ca-Alr4331N, but for expressing RNase E N-terminal variant having the point mutation N141R	this study
pUT18Ca-Alr4331N^{A145R}	Car^r; same as pUT18Ca-Alr4331N, but for expressing RNase E N-terminal variant having the point mutation A145R	this study
pUT18Ca-Alr4331N^{L146R}	Car^r; same as pUT18Ca-Alr4331N, but for expressing RNase E N-terminal variant having the point mutation L146R	this study
pUT18Ca-Alr4331N^{I148R}	Car^r; same as pUT18Ca-Alr4331N, but for expressing RNase E N-terminal variant having the point mutation I148R	this study
pUT18Ca-Alr4331N^{K151E}	Car^r; same as pUT18Ca-Alr4331N, but for expressing RNase E N-terminal variant having the point mutation K151E	this study
pKT25a-Alr4331(1-117)	Km^r; pKT25 derivate encoding RNase E N-terminal region (1-117 aa, the S1 domain) fused to the C-terminal of T25.	this study
pUT18Ca-Alr4331(1-117)	Car^r; pUT18Ca derivate encoding RNase E N-terminal region (1-117 aa, the S1 domain) fused to the C-terminal of T18.	this study
pKT25a-Alr4331(100-210)	Km^r; pKT25 derivate encoding RNase E N-terminal region (100-210 aa, the 5' sensor domain) fused to the C-terminal of T25.	this study
pUT18Ca-Alr4331(100-210)	Car^r; pUT18Ca derivate encoding RNase E N-terminal region (100-210 aa, the 5' sensor domain) fused to the C-terminal of T18.	this study
pKT25a-Alr4331(196-273)	Km^r; pKT25 derivate encoding RNase E N-terminal region (196-273 aa, the RNase H domain) fused to the C-terminal of T25.	this study
pUT18Ca-Alr4331(196-273)	Car^r; pUT18Ca derivate encoding RNase E N-terminal region (196-273 aa, the RNase H domain) fused to the C-terminal of T18.	this study
pKT25a-Alr4331(255-396)	Km^r; pKT25 derivate encoding RNase E N-terminal region (255-396 aa, the DNase I domain) fused to the C-terminal of T25.	this study
pUT18Ca-Alr4331(255-396)	Car^r; pUT18Ca derivate encoding RNase E N-terminal region (225-396 aa, the DNase I domain) fused to the C-terminal of T18.	this study
pKT25a-All4396	Km^r; pKT25 derivate encoding PNPase fused to the C-terminal of T25.	this study
pUT18Ca-All4396	Car^r; pUT18Ca derivate encoding PNPase fused to the C-terminal of T18.	this study
pKT25a-Alr1240	Km^r; pKT25 derivate encoding RNase II fused to the C-terminal of T25.	9

pUT18Ca-Alr1240	Car[r]; pUT18Ca derivate encoding RNase II fused to the C-terminal of T18.	9
pKT25a-Alr1223	Km[r]; pKT25 derivate encoding CrhB fused to the C-terminal of T25.	this study
pUT18Ca-Alr1223	Car[r]; pUT18Ca derivate encoding CrhB fused to the C-terminal of T18.	this study
PpKT25a-EcRneN529	Km[r]; pKT25 derivate encoding *E. coli* RNase E N-terminal region (1-529 aa) fused to the C-terminal of T25.	this study
pUT18Ca-EcRneN529	Car[r]; pUT18Ca derivate encoding *E. coli* RNase E N-terminal region (1-529 aa) to the C-terminal of T18.	this study

Table S3. Oligonucleotides used in this study.

Name	Sequence (5'-3')	Description
**for construction of cyanobacterial plasmids**		
Pall1338F1005m	GCAGAAATTCGATATCTAGATCGTACTGAGTCCTGAGGTAGAAGTCA	to amplify a region upstream *rebA* ORF, which is used to construct the plasmid pCpf1b-ΔrebA
Pall1338R11	AGCGCTACCGACGCTAGTGCTTTGTTAGTTTTCTCACGGTAGATAC	
Pall1338R2225	CGCAACGTTGTTGCCATTGCAGGAACTTGGTCTGATGACAG	to amplify a region downstream *rebA* ORF, which is used to construct the plasmid pCpf1b-ΔrebA
Pall1338F1247a	GCACTAGCGTCGGTAGCGCTCAGGTCTATTCTTTGTCTCTGGTGTA	
cr_all1338R1130F	AGATGCCACACGGTGGTTATCAAATA	to generate the DNA duplex of a spacer sequence, which is used to construct the plasmid pCpf1b-ΔrebA
cr_all1338R1130R	AGACTATTTGATAACCACCGTGTGGC	
Pall1340F681	TGAGGAGTTATTTGCTAGTGCCAGTAACTATTGGCAGTTGCGAG	to verify the genotype of Δ*rebA* strain
Pall1338R2225	CGCAACGTTGTTGCCATTGCAGGAACTTGGTCTGATGACAG	
Pall1338R797	GGTCTTGCGATTACCTGCGGTGGAGCA	
P25TNotI-F	AAGCCCAAGCGTTTTGTTATTGG	to amplify the vector fragment from pCT, which is used to construct the plasmid pPrbcL-RebA
P25TBamHI-R	GGATCCAAAAAAAAACCCCGCCGAAGC	
PPrbcL-F	CGGGGTTTTTTTTTGGATCCGCAGGGGAAGTAAAGAAGAATGA	to amplify the promoter of PrbcL from *Anabaena* genomic DNA, which is used to construct the plasmid pPrbcL-RebA
PPrbcL-R	CTCGAGTATCCTTCCAAGATGTC	
Pall1338bF1c	CTTGGAAGGATACTCGAGATGCGCAAACTGACCAACAGCGACAA	to amplify the *rebA* gene from pNStrep-RebA, which is used to construct the plasmid pPrbcL-RebA
Pall1338bR1272c	AACAAAACGCTTGGGCTTTTACACCAGGCTCAAGGAGTAGACTT	
PAll1338bV337A_f	ACCTGGCCATTGATCGTAGCTCCGAACTG	to generate the mutation V337A in RebA by site-directed mutagenesis, using the template of pPrbcL-RebA or pNStrep-RebA.
PAll1338bV337A_r	TCAATGGCCAGGTAGCAGGTTTTCGGCA	
PAll1338bL368A_f	CCGCGCCGATTTTCGACAACCACCGT	same as above, but to generate the mutation L368A in RebA.
PAll1338bL368A_r	AAAATCGGCGCGGTGCGCTGCTGGGT	
PAll1338bI370A_f	GCCGGCGTTCGACAACCACCGTGTG	same as above, but to generate the mutation I370A in RebA.
PAll1338bI370A_r	GTCGAACGCCGGCAGGGTGCGCTG	
PAll1338bV387A_f	GCGCAATTAAGGTTCCGGATAGCCGTA	same as above, but to generate the mutation V387A in RebA.
PAll1338bV387A_r	ACCTTAATTGCGCGGTCACGCTTGGTG	
PAll1338bI388A_f	GTTGCGAAGGTTCCGGATAGCCGTAT	same as above, but to generate the mutation I337A in RebA.
PAll1338bI388A_r	GAACCTTCGCAACGCGGTCACGCTTG	

Palr4331F1596m	GCAGAAATTCGATATCTAGATGCGATCGCCCAAGCCGGTCTA	to amplify a region upstream *rne* ORF, which is used to construct the plasmid pICT-rne.
Palr4331R556m	GGCTCGACTCTAGCTAGAGTTATACAGCTAAAATTAGTTGATTGCGA	
PcoquwF	CTCTAGCTAGAGTCGAGCCCGTTAAGGGATTTTGGTCATGAG	to amplify the CT promoter from the plasmid pCT, which is used to construct the plasmid pICT-rne
PV_19	CATCTTGTTGTTACCTCCTTAGCA	
Palr4331F1b	TGCTAAGGAGGTAACAACAAGATGCCAAAACAAATTATTATCGCGG	to amplify a 5' region of *rne* ORF, which is used to construct the plasmid pICT-*rne*
Palr4331R990	CGCAACGTTGTTGCCATTGCCCGCAGACGTAGCTGGCGAGCA	
cr_alr4331F26mF	AGATATACTGCCGATTTTTGAGGAAA	to generate the DNA duplex of a spacer sequence, which is used to construct the plasmid pICT-*rne*.
cr_alr4331F26mR	AGACTTTCCTCAAAAATCGGCAGTAT	
Pall0258F475m	CTTGGATCCTAAAGCCTGTGAAATTAACTG	to verify the genotype of the strain CT-*rne*.
Palr4331R1020	GAAATCAACGACGATTACCCCAGCGAT	
Palr4331F1596m	GCAGAAATTCGATATCTAGATGCGATCGCCCAAGCCGGTCTA	
Palr4331R33m	AAGGGCATCAAGACGATGCTGGTATCACCGCTTGGAGGTTTGTTATCCCT	
Pall3854F219ma	ATCCTAATACGACTCACTATAGATTTCCCTAATAAAGCAAGCGGCTGTAT	to amplify the DNA template for *in vitro* transcription of *psbO* mRNA by T7 RNA polymerase.
Pall3854R1095	AATAAAAAAAGGGCTAGAAGCCCCTTTCTGTGATT	
Palr4866F65maa	ATCCTAATACGACTCACTATAGACAGGCGCAAGTCTAAAAAAAGCGTCTGAATTA	to amplify the DNA template for in vitro transcription of *psbAI* mRNA by T7 RNA polymerase.
Palr4866R1144a	TAAAAAAGCGCTCTTCCATGCAAATTGGAAGGGCGCTTTTTTAGTTAACTAAAACTGA	
PV_19	CATCTTGTTGTTACCTCCTTAGCA	to amplify the vector fragment from pCT, which is used to construct the plasmid pCT-SynRebA and pCT-rnhB.
PV_26	GGTGGATCTGGAGGTAGTGGTTCTGGTGGTGGTAGCAC	
Psyn7942-rebAF1	TAAGGAGGTAACAACAAGATGGCCCCTGCCAAGCTTTCTCCTGCTGATA	to amplify the homolog gene of *rebA* from *Synechococcus elongatus* PCC 7942 genomic DNA, which is used to construct the plasmid pCT-SynRebA.
Psyn7942-rebAR1122	ACCACTACCTCCAGATCCACCGCGATCCAGCGCGTAGACCTGACCAT	
Palr4332F1m	TAAGGAGGTAACAACAAGATGATGCTAAAGAAAGAGCAAAC	to amplify the *rnhB* ORF, which is used to construct the plasmid pCT-RnhB.
Palr4332R675	ACCACTACCTCCAGATCCACCGTCAGGTGATATTTGACAAGGG	
Palr4332F797m	TGGCAGAAATTCGATATCTAGATCAGTGAGTCAGATTTAGACCTTGATGG	to amplify a region upstream *rnhB* ORF, which is used to construct the plasmid pCpf1b-ΔrnhB.
Palr4332R1m	CAAAGCTTACACTAATACACAATATCTCA	
Palr4332F679	TGTATTAGTGTAAGCTTTGTTAATTCGTAATTAAACTGTTCTTTTCTTTCAACA	

Palr4332R1464	CGCAACGTTGTTGCCATTGCAACTAAGTGGCATTTAGCCCATCAACTA	to amplify a region downstream *rnhB* ORF, which is used to construct the plasmid pCpf1b-ΔrnhB.
cr_alr4332R379F	AGATGCTTTTTTACAGACCGTTTCAT	to generate the DNA duplex of a spacer sequence, which is used to construct the plasmid pCpf1b-ΔrnhB.
cr_alr4332R379R	AGACATGAAACGGTCTGTAAAAAAGC	
Palr4332F928m	TAATCATCCTAGCTATCAAGAACTGAAC	to verify the genotype of the strain Δ*rnhB.*
Palr4332R586	CATAGCCCTTGTTACTCTTTAAGTCATACA	
Palr4332F179	GCCTGTAGTGGCAGCATCTGTCATATTACC	
Palr4332R1538	ATGAGCTATAATCTATATACTTACGAACCA	
**for construction of protein expression plasmids**		
PV_1	GCTGCCGCGCGGCACCAG	to amplify the vector fragment from pET28a, which is used to construct the plasmid pHT-EcRneN529.
PV_2	CTGAGATCCGGCTGCTAAC	
Pb1084F1	CTGGTGCCGCGCGGCAGCATGAAAAGAATGTTAATCAACGCAACTCAGCAG GAAGAG	to amplify a region of *E. coli rne* gene, which is used to construct the plasmid pHT-EcRneN529.
Pb1084R1587	GTTAGCAGCCGGATCTCAGTTACAGCGCAGGTTGTTCCGGACGCTTACGTT CA	
PV_5	TGCACCCTTTTCGAACTGC	to amplify the vector fragment from pNStrep, which is used to construct the plasmids pNStrep-Alr4331N396 and pNStrep-RebA.
PV_2	CTGAGATCCGGCTGCTAAC	
Palr4331F4b	CAGTTCGAAAAGGGTGCAATGCCAAAACAAATTATTATCGCGGAGCA	to construct the plasmid pNStrep-Alr4331N396.
Palr4331R1188b	GTTAGCAGCCGGATCTCAGTTAAGTATCACCAAACAATTCATAAATATTTTGCC CT	
Pall1338F901	AACAGGTACCCATATGGACGACTTAGAGGAGGCAAGACC	to amplify a *rebA* region from Anabaena chromosome, which is used to construct the plasmid pHS-RebA_C.
Pall1338R1269	TTCTACTCGAGTCACCAGAGACAAAGAATAGACCT	
Pall1338F2	AACAGGTACCCATATGAGAAAACTAACAAACTCTG	to amplify a *rebA* region from Anabaena chromosome, which is used to construct the plasmid pHS-RebA_DC.
Pall1338R960	AATCTCGAGTCACTGCGGCATCCCCAAATC	
PAlr4331A145R_f	GGCGTTTAGCAATTTTAATTAAACCTGCCGG	to amplify pHTAlr4331N412 for constructing pHTAlr4331N412^{A145R}
PAlr4331A145R_r	ATTGCTAAACGCCGTAAGCGGTTACGTTCAC	
PAlr4331I148R_f	CACGTTTAATTAAACCTGCCGGCATGGGT	to amplify pHTAlr4331N412 for constructing pHTAlr4331N412^{I148R}
PAlr4331I148R_r	TTAATTAAACGTGCTAATGCCCGTAAGCG	

PAlr4331K151E_f	AATTTTAATTGAGCCTGCCGGCATGGGT	to amplify pHTAlr4331N412 for constructing pHTAlr4331N412^{K151E}
PAlr4331K151E_r	CTCAATTAAAATTGCTAATGCCCGTAAG	
**for construction of bacterial two-hybrid assay plasmids**		
PpKT25a-F733	GAGATCTAGATCGACATCTGTCGAGC	to prepare the linear fragment of the vector pKT25a or pUT18C.
PpKT25a-R720	TTCACCACTAGAGGTGATCA	
Pall1338F1b	ATCACCTCTAGTGGTGAAGTGAGAAAACTAACAAACTCTGACAA	to amplify the *Anabaena* genomic region encoding RebA (2-423 aa)
Pall1338R1272a	GATGTCGATCTAGATCTCTTACACCAGAGACAAAGAATAGACCT	
Pall1338bF4	ATCACCTCTAGTGGTGAACGCAAACTGACCAACAGCGACAAGCAGGA	to amplify the *Anabaena* genomic region encoding RebA (2-423 aa).
Pall1338bR1272b	GATGTCGATCTAGATCTCTTACACCAGGCTCAAGGAGTAGACTT	
Palr4331F1c	ATCACCTCTAGTGGTGAAATGCCAAAACAAATTATTATCGCGG	to amplify the *Anabaena* genomic region encoding RNase E (1-688 aa).
Palr4332R2094	GATGTCGATCTAGATCTCAATCTGTGGCTCTGCTTCG	
Palr4331F4	ATCACCTCTAGTGGTGAACCAAAACAAATTATTATCGCGGAGCAGCATC	to amplify the *Anabaena* genomic region encoding RNase E N terminal domain (2-396 aa).
Palr4331R1188	GATGTCGATCTAGATCTCTTAAGTATCACCAAACAATTCATAAATATTTTGCCC T	
PpKT25a-F733	GAGATCTAGATCGACATCTGTCGAGC	to amplify pKT25a-Alr4331N or pUT18Ca-Alr4331N for constructing pKT25a-Alr4331 (2-117) or pUT18Ca-Alr4331 (2-117)
Palr4331R351	GTCGATCTAGATCTCTTAACGTCCAGGCAGAGTGATATTACCTGTGAG	
PAlr4331N141R_f	GTGAACGTCGTCGCTTACGGGCATTAGCAA	To amplify pUT18Ca-Alr4331N for constructing pUT18Ca-Alr4331N^{N141R}
PAlr4331N141R_r	CGACGACGTTCACTCTCACTCTTAATCCG	
PAlr4331A145R_f	GGCGTTTAGCAATTTTAATTAAACCTGCCGG	To amplify pUT18Ca-Alr4331N for constructing pUT18Ca-Alr4331N^{A145R}
PAlr4331A145R_r	ATTGCTAAACGCCGTAAGCGGTTACGTTCAC	
PAlr4331L146R_f	GCACGCGCAATTTTAATTAAACCTGCCGG	To amplify pUT18Ca-Alr4331N for constructing pUT18Ca-Alr4331N^{L146R}
PAlr4331L146R_r	AAATTGCGCGTGCCCGTAAGCGGTTACG	
PAlr4331I148R_f	CACGTTTAATTAAACCTGCCGGCATGGGT	To amplify pUT18Ca-Alr4331N for constructing pUT18Ca-Alr4331N^{N148R}
PAlr4331I148R_r	TTAATTAAACGTGCTAATGCCCGTAAGCG	
PAlr4331K151E_f	AATTTTAATTGAGCCTGCCGGCATGGGT	To amplify pUT18Ca-Alr4331N for constructing pUT18Ca-Alr4331N^{K151E}
PAlr4331K151E_r	CTCAATTAAAATTGCTAATGCCCGTAAG	
Palr4331F298	ATCACCTCTAGTGGTGAACCAACGGGAACAAAAGGCCCAAGGCTCACA	to amplify the *Anabaena* genomic region encoding RNase E N terminal domain (100-210 aa).
Palr4331R630	GATGTCGATCTAGATCTCACGTAGTACGCGCTGGATAAAGTCAT	
Palr4331F586	ATCACCTCTAGTGGTGAAGCACTCCTGAATCGGGACGATGACTTTATCC	

Palr4331R819	GATGTCGATCTAGATCTCTTTAAGGGCTTCTCGAATTGCGGCATTGA	to amplify the *Anabaena* genomic region encoding RNase E N terminal domain (196-273 aa).
Pall1338F901	ATCACCTCTAGTGGTGAAGACGACTTAGAGGAGGCAAGACCTCTAGTTAC	to amplify the *Anabaena* genomic region encoding RebA C terminal domain (300-424 aa).
Pall1338R1272a	GATGTCGATCTAGATCTCTTACACCAGAGACAAAGAATAGACCT	
Palr4331F763	ACCTCTAGTGGTGAACGCTCCCCAATTTTAGAATATTTCC	to amplify pKT25a-Alr4331N or pUT18Ca-Alr4331N for constructing pKT25a-Alr4331 (255-396 aa) or pUT18Ca-Alr4331 (255-396 aa)
PpKT25a-R720	TTCACCACTAGAGGTGATCA	
PAll1338_C334S_f	ACCTCATATTTGGTTATTGATCGCTCCTCG	to create the C334S mutation in RebA in the two-hybrid plasmids of RebA (i.e., pKT25-RebA and pUT18Ca-RebA).
PAll1338_C334S_r	CCAAATATGAGGTCTTGGGCAAAGATGCC	
PAll1338_Y335A_f	CTGCGCATTGGTTATTGATCGCTCCTCG	same as above, but for the mutation Y335A
PAll1338_Y335A_r	AACCAATGCGCAGGTCTTGGGCAAAGAT	
PAll1338_L336A_f	GCTATGCAGTTATTGATCGCTCCTCGGAG	same as above, but for the mutation L336A
PAll1338_L336A_r	ATAACTGCATAGCAGGTCTTGGGCAAAGA	
PAll1338_V337A_f	ATTTGGCCATTGATCGCTCCTCGGAGTTA	same as above, but for the mutation V337A
PAll1338_V337A_r	TCAATGGCCAAATAGCAGGTCTTGGGCAA	
PAll1338_I338A_f	GGTTGCAGATCGCTCCTCGGAGTTAATTAC	same as above, but for the mutation I338A
PAll1338_I338A_r	GCGATCTGCAACCAAATAGCAGGTCTTGGG	
PAll1338_D339A_f	GGTTATTGCACGCTCCTCGGAGTTAATTACC	same as above, but for the mutation D339A
PAll1338_D339A_r	GCGTGCAATAACCAAATAGCAGGTCTTGGG	
PAll1338_E343A_f	TCGGCTTTAATTACCAGACCACTCAAAGACTT	same as above, but for the mutation E343A
PAll1338_E343A_r	TAATTAAAGCCGAGGAGCGATCAATAACCAAA	
PAll1338_L344A_f	GAGGCCATTACCAGACCACTCAAAGACTTTG	same as above, but for the mutation L344A
PAll1338_L344A_r	TGGTAATGGCCTCCGAGGAGCGATCAATAAC	
PAll1338_T346A_f	TAATTGCAAGACCACTCAAAGACTTTGGTG	same as above, but for the mutation T346A
PAll1338_T346A_r	GGTCTTGCAATTAACTCCGAGGAGCGATCA	
PAll1338_R347A_f	TACCGCTCCACTCAAAGACTTTGGTGATTTG	same as above, but for the mutation R347A
PAll1338_R347A_r	GAGTGGAGCGGTAATTAACTCCGAGGAGCGA	
PAll1338_L349A_f	AGACCAGCGAAAGACTTTGGTGATTTGGGGC	same as above, but for the mutation L349A
PAll1338_L349A_r	CTTTCGCTGGTCTGGTAATTAACTCCGAGG	
PAll1338_D351A_f	CTCAAAGCCTTTGGTGATTTGGGGCAAATTCC	same as above, but for the mutation D351A
PAll1338_D351A_r	CAAAGGCTTTGAGTGGTCTGGTAATTAACTCC	
PAll1338_F352A_f	CAAAGACGCCGGTGATTTGGGGCAAATTCCTA	same as above, but for the mutation F352A

PAll1338_F352A_r	ACCGGCGTCTTTGAGTGGTCTGGTAATTAACT	
PAll1338_L361A_f	CCTAGTGCAGAAACCCAGCAAAGAACACTAC	same as above, but for the mutation L361A
PAll1338_L361A_r	TTTCTGCACTAGGAATTTGCCCCAAATCAC	
PAll1338_E362A_f	AGTCTAGCCACCCAGCAAAGAACACTACCA	same as above, but for the mutation E362A
PAll1338_E362A_r	GGGTGGCTAGACTAGGAATTTGCCCCAAAT	
PAll1338_T363A_f	GAAGCGCAGCAAAGAACACTACCAATATTTGAT	same as above, but for the mutation T363A
PAll1338_T363A_r	TTTGCTGCGCTTCTAGACTAGGAATTTGCCCCA	
PAll1338_T367A_f	AAAGAGCACTACCAATATTTGATAACCACCGTG	same as above, but for the mutation T367A
PAll1338_T367A_r	GGTAGTGCTCTTTGCTGGGTTTCTAGACTAGG	
PAll1338_L368A_f	GAACAGCGCCAATATTTGATAACCACCGTGTG	same as above, but for the mutation L368A
PAll1338_L368A_r	ATTGGCGCTGTTCTTTGCTGGGTTTCTAGAC	
PAll1338_I370A_f	CTACCAGCTTTTGATAACCACCGTGTGGC	same as above, but for the mutation I370A
PAll1338_I370A_r	CAAAAGCTGGTAGTGTTCTTTGCTGGGT	
PAll1338_F371A_f	CCAATAGCTGATAACCACCGTGTGGCTAAA	same as above, but for the mutation F371A
PAll1338_F371A_r	TATCAGCTATTGGTAGTGTTCTTTGCTGGG	
PAll1338_F380A_f	ACGCGCGTCTACCAAGCGCGATCGCGT	same as above, but for the mutation F380A
PAll1338_F380A_r	GGTAGACGCGCGTTTAGCCACACGGT	
PAll1338_S381A_f	CGCTTTGCCACCAAGCGCGATCGCGT	same as above, but for the mutation S381A
PAll1338_S381A_r	TGGTGGCAAAGCGTTTAGCCACACGG	
PAll1338_D385A_f	AGCGCGCACGCGTGATTAAAGTCCCCG	same as above, but for the mutation D385A
PAll1338_D385A_r	ACGCGTGCGCGCTTGGTAGAAAAGCGT	
PAll1338_R386A_f	ATGCTGTGATTAAAGTCCCCGATAGTAGAAT	same as above, but for the mutation R386A
PAll1338_R386A_r	TTAATCACAGCATCGCGCTTGGTAGAAAAGC	
PAll1338_V387A_f	CGCTATTAAAGTCCCCGATAGTAGAATGC	same as above, but for the mutation V387A
PAll1338_V387A_r	GACTTTAATAGCGCGATCGCGCTTGGTAG	
PAll1338_I388A_f	TGGCCAAAGTCCCCGATAGTAGAATGCT	same as above, but for the mutation I388A
PAll1338_I388A_r	GGGACTTTGGCCACGCGATCGCGCTTG	
PAll1338_K389A_f	ATTGCGGTCCCCGATAGTAGAATGCTG	same as above, but for the mutation K389A
PAll1338_K389A_r	CGGGGACCGCAATCACGCGATCGCGCT	
PAll1338_V390A_f	TTAAAGCCCCCGATAGTAGAATGCTGCAC	same as above, but for the mutation V390A
PAll1338_V390A_r	TCGGGGGCTTTAATCACGCGATCGCGCT	
PAll1338_M395A_f	GATAGTAGAGCGCTGCACAAAGCTCGCACC	same as above, but for the mutation M395A
PAll1338_M395A_r	GCGCTCTACTATCGGGGACTTTAATCACG	
PAll1338_L396A_f	AGTAGAATGGCTCACAAAGCTCGCACCCAT	same as above, but for the mutation L396A
PAll1338_L396A_r	GAGCCATTCTACTATCGGGGACTTTAATCA	

PAll1338_L403A_f	CATGCCCAAGCTAAGGGTATCACCAGAC	same as above, but for the mutation L403A
PAll1338_L403A_r	TAGCTTGGGCATGGGTGCGAGCTTTGTG	

Supplementary References

1. Shih,P.M., Wu,D., Latifi,A., Axen,S.D., Fewer,D.P., Talla,E., Calteau,A., Cai,F., Tandeau de Marsac,N. and Rippka,R. et al. (2013) Improving the coverage of the cyanobacterial phylum using diversity-driven genome sequencing. *Proc. Natl. Acad. Sci. USA*, **110**, 1053-1058.
2. Kumar,S., Stecher,G., Li,M., Knyaz,C. and Tamura,K. (2018) MEGA x: molecular evolutionary genetics analysis across computing platforms. *Mol. Biol. Evol.*, **35**, 1547-1549.
3. Saitou,N. and Nei,M. (1987) The neighbor-joining method: a new method for reconstructing phylogenetic trees. *Mol. Biol. Evol.*, **4**, 406-425.
4. Felsenstein,J. (1985) CONFIDENCE LIMITS ON PHYLOGENIES: AN APPROACH USING THE BOOTSTRAP. *Evolution.*, **39**, 783-791.
5. Robert,X. and Gouet,P. (2014) Deciphering key features in protein structures with the new ENDscript server. *Nucleic Acids Res.*, **42**, W320-4.
6. Mitschke,J., Vioque,A., Haas,F., Hess,W.R. and Muro-Pastor,A.M. (2011) Dynamics of transcriptional start site selection during nitrogen stress-induced cell differentiation in *Anabaena* sp. PCC7120. *Proc. Natl. Acad. Sci. USA*, **108**, 20130-20135.
7. Battesti,A. and Bouveret,E. (2012) The bacterial two-hybrid system based on adenylate cyclase reconstitution in *Escherichia coli*. *Methods.*, **58**, 325-334.
8. Elhai,J., Vepritskiy,A., Muro-Pastor,A.M., Flores,E. and Wolk,C.P. (1997) Reduction of conjugal transfer efficiency by three restriction activities of *Anabaena* sp. strain PCC 7120. *J. Bacteriol.*, **179**, 1998-2005.
9. Zhou,C., Zhang,J., Hu,X., Li,C., Wang,L., Huang,Q. and Chen,W. (2020) RNase II binds to RNase E and modulates its endoribonucleolytic activity in the cyanobacterium *Anabaena* PCC 7120. *Nucleic Acids Res.*, **48**, 3922-3934.
10. Niu,T.C., Lin,G.M., Xie,L.R., Wang,Z.Q., Xing,W.Y., Zhang,J.Y. and Zhang,C.C. (2019) Expanding the potential of CRISPR-Cpf1-Based genome editing technology in the cyanobacterium *Anabaena* PCC 7120. *ACS Synth. Biol.*, **8**, 170-180.
11. Zhang,J.Y., Deng,X.M., Li,F.P., Wang,L., Huang,Q.Y., Zhang,C.C. and Chen,W.L. (2014) RNase E forms a complex with polynucleotide phosphorylase in cyanobacteria via a cyanobacterial-specific nonapeptide in the noncatalytic region. *RNA*, **20**, 568-579.
